# Cardiovascular Complications in Patients with Prostate Cancer: Potential Molecular Connections

**DOI:** 10.3390/ijms24086984

**Published:** 2023-04-10

**Authors:** Sooraj Kakkat, Paramahansa Pramanik, Seema Singh, Ajay Pratap Singh, Chandrani Sarkar, Debanjan Chakroborty

**Affiliations:** 1 Department of Pathology, University of South Alabama, Mobile, AL 36617, USA; 2 Cancer Biology Program, Mitchell Cancer Institute, University of South Alabama, Mobile, AL 36604, USA; 3 Department of Mathematics and Statistics, University of South Alabama, Mobile, AL 36688, USA; 4 Department of Biochemistry and Molecular Biology, University of South Alabama, Mobile, AL 36688, USA

**Keywords:** prostate cancer, cardiovascular diseases, androgen deprivation therapy

## Abstract

Cardiovascular diseases (CVDs) and complications are often seen in patients with prostate cancer (PCa) and affect their clinical management. Despite acceptable safety profiles and patient compliance, androgen deprivation therapy (ADT), the mainstay of PCa treatment and chemotherapy, has increased cardiovascular risks and metabolic syndromes in patients. A growing body of evidence also suggests that patients with pre-existing cardiovascular conditions show an increased incidence of PCa and present with fatal forms of the disease. Therefore, it is possible that a molecular link exists between the two diseases, which has not yet been unraveled. This article provides insight into the connection between PCa and CVDs. In this context, we present our findings linking PCa progression with patients’ cardiovascular health by performing a comprehensive gene expression study, gene set enrichment (GSEA) and biological pathway analysis using publicly available data extracted from patients with advanced metastatic PCa. We also discuss the common androgen deprivation strategies and CVDs most frequently reported in PCa patients and present evidence from various clinical trials that suggest that therapy induces CVD in PCa patients.

## 1. Introduction

Prostate cancer (PCa) is the most prevalent non-cutaneous malignancy among men in the United States (US). It is estimated that there will be 288,300 new cases of PCa and about 34,700 cancer-related deaths in 2023 [1]. When detected early, the disease might be cured by radiotherapy or surgery, with a 15-year survival rate above 90%. However, in spite of improvements in treatment strategies, a disease relapse has been noted in 30–60% of the patients [2,3], and treatment outcomes for the metastatic forms of the disease are not very promising. Androgen deprivation therapy (ADT) remains the cornerstone of treatment for metastatic PCa [4,5,6,7], but most patients experience disease relapse due to therapeutic failure. Several studies in recent times have suggested a strong connection between cardiovascular diseases (CVDs) and PCa [8,9,10,11,12,13,14]. ADT reportedly elevates the risk for heart failure and other CVDs like hypertension and stroke in men without pre-existing cardiovascular conditions, which is even more in patients with pre-existing CVDs [8,9,10,11,12,13,14]. Indeed, cardiovascular complications are the most common cause of death in PCa patients [15,16,17]. Interestingly, in some reports, CVDs have also been shown to be associated with increased PCa risk [18,19,20,21], more advanced PCa at diagnosis [22] and higher PCa-related mortalities [18,23]. Despite these reports, a mechanistic link between PCa and CVDs has not yet been established. Here, we review common CVDs associated with PCa and their impact on disease management. In addition, we also present a comprehensive analysis of publicly available transcriptomic data from PCa patients and identify the most abundant biological pathways via gene set enrichment and pathway analyses to suggest a molecular connection of PCa with adverse cardiovascular events.

## 2. Androgen Deprivation Therapy and Cardiovascular Complications

Androgen and androgen receptors (AR) play integral roles in the development and homeostasis of the normal prostate [24]. The androgen axis also plays an important role in PCa development and progression. Huggins and Hodges first reported the clinical effects of suppression of serum testosterone on PCa progression in 1941. Since then, ADT has remained a major treatment strategy in PCa [25,26,27]. However, cardiovascular complications have been recognized as major adverse events (AE) with ADT and treatment-related adverse effects have been cited as a major reason for the deaths of PCa patients. The probable reason for therapy-related CVD could be that testosterone plays an important role in the cardiovascular system; therefore, the suppression of testosterone in ADT leads to the malfunction of the system [28]. Testosterone has a profound effect on vasculature and causes vasodilation, both in endothelium dependent and independent manners [29,30]. Low testosterone level in men is associated with adverse cardio-metabolic factors like increased inflammation, dyslipidemia, insulin resistance and atherosclerosis [30]. Higher endogenous testosterone level in patients is associated with reduced cardiovascular risks and CVD-related mortality compared to patients deficient in testosterone and patients who have undergone treatment with testosterone replacement therapy. Moreover, testosterone replacement therapy has been reported to improve myocardial ischemia in men who have coronary artery diseases (CAD) and exercise capacity in men with congestive heart failure (CHF) [28]. Therefore, it is possible that androgen inhibition in PCa resulting from ADT, which reduces the testosterone level, also affects the patient’s cardiovascular health in a negative manner. The most significant association between CVDs and ADT comes from observational studies where ADTs like orchiectomy (surgical removal of testis) and treatment with gonadotropin receptor hormone (GnRH) agonists and antagonists (first line systemic agents for ADT) were reported to promote adverse cardiovascular events in patients [31,32,33,34]. Both orchiectomy and GnRH agonists and antagonists reduce hormones such as luteinizing hormone (LH) and follicle-stimulating hormone (FSH), which eventually decrease the level of testosterone in patients [31]. Studies show a strong association between GnRH agonist use and the increased incidence of cardiovascular events in patients with PCa. Increased diabetes and cardiovascular events like CAD, myocardial infarction and sudden cardiac deaths were noted among patients receiving GnRH agonists as ADT compared to patients not receiving any ADT [10]. GnRH agonists also affect cardiometabolic conditions like increased body fat mass and the development of insulin resistance in PCa patients [35]. A large cohort study consisting of 14,375 PCa patients treated either with GnRH agonists or bilateral orchiectomy reported that both types of ADT had a similar impact on patients’ cardiovascular health [36]. Other studies reported that bilateral orchiectomy is associated with the development of metabolic conditions like diabetes that indirectly affect patients’ cardiovascular conditions rather than directly causing cardiovascular complications like CAD or myocardial infarction, or sudden cardiac arrests [35]. In the PRONOUNCE randomized trial conducted to compare the cardiovascular safety of degarelix, a GnRH receptor antagonist, with leuprolide, a GnRH receptor agonist, in patients with advanced PCa, a major adverse cardiovascular event was noted in 15 (5.5%) patients belonging to the degarelix group and 11 (4.1%) patients in the leuprolide group (hazard ratio, 1.28 [95% CI, 0.59–2.79]; *p* = 0.53) [37,38,39]. In the HERO trial determining the efficacy and safety of relugolix, an oral gonadotropin-releasing hormone antagonist, compared to leuprolide, it was reported that the incidence of major adverse cardiovascular events in the relugolix group was 2.9% as compared to 6.2% in the leuprolide group (hazard ratio, 0.46; 95% CI, 0.24 to 0.88) [40,41]. The results from the PRONOUNCE and HERO trials have been summarized in Table 1.

New generation ADTs like abiraterone acetate and enzalutamide directly inhibit androgen synthesis or androgen functions via inhibition of AR, leading to low testosterone levels or lesser action, which increases the risk of developing CVDs like coronary artery disease, congestive heart failure (CHF) and metabolic syndromes like diabetes [31]. Moreover, PCa generally strongly correlates with many risk factors associated with CVDs, like obesity, hypertension, smoking, and diet [31]. Although the relation between PCa and CVDs is not completely understood, reports indicate that PCa patients receiving ADT experience CVDs, which is even more in patients with a prior history of CVD.

## 3. Cardiovascular Diseases and Prostate Cancer

Keating and colleagues first reported the increased risk of developing CVDs like coronary heart diseases (CHD), myocardial infarction (MI), hypertension, stroke, arrhythmia, and sudden cardiac arrests in PCa patients with the use of GnRH agonists for treatment [10,42]. Thereafter, subsequent studies reported the association between androgen inhibition and cardiovascular events, even in patients receiving second-generation androgen inhibitors. This section will discuss the most common cardiovascular events observed in PCa patients.

*Hypertension*: Hypertension or high blood pressure plays a critical role in disease progression and is the major cardiovascular event experienced by the majority of patients with metastatic castration-resistant prostate cancer (mCRPC). Reports indicate that hypertension not only increases the risk of PCa development [18,19,20,21] but also patients who have hypertension are presented with more advanced forms of PCa at diagnosis [22] and are more likely to succumb to PCa [18,23]. Several studies, including a recent meta-analysis of data, show that patients taking medications for hypertension have a lesser incidence of PCa in their lifetime [18,19,20,21]. In a recent study performed on the Swedish patient population, it was reported that patients with hypertension have more fatal forms of PCa compared to patients who do not have hypertension [43]. A consistent association between the development of hypertension and the use of second-generation androgen inhibitors, abiraterone, and enzalutamide, has been reported [14,44]. Although the mechanism is not completely understood for AR antagonists like enzalutamide, compounds like abiraterone inhibit androgen biosynthesis by the continuous and irreversible inhibition of 17 alpha-hydroxylase/C17, 20 lyase (CYP17A1) [45]. Inhibition of CYP17A1 results in decreased plasma cortisol in patients, increasing the production of adrenocorticotropic hormones (ACTH) and eventually promoting the development of hypertension [46]. In addition, ADT has been reported to increase arterial stiffness in patients, which might result in higher blood pressure [47].

*Coronary arterial diseases (CADs)*: CADs, caused by excessive plaque buildup in major arteries supplying blood to the heart, are major cardiovascular events associated with PCa. Diseases like hypertension that put a strain on the heart are known to cause CADs. In addition, conditions such as high cholesterol, high lipoprotein, thrombosis, and metabolic conditions like diabetes have been cited as major reasons for CADs in patients. Both high and low-grade PCa show similar associations with CADs [48]. Also, in patients receiving ADT, CADs have been cited as major treatment-related toxicities, often leading to premature death or discontinuation of the treatment. Although the potential mechanism by which CADs elevate the risk of PCa is unclear, a myriad of risk factors are shared between the two diseases. One such risk factor is the plasma cholesterol level. Several studies have reported that high plasma cholesterol leads to CADs, which increases the risk of PCa [48,49,50,51]. Furthermore, a significant number of genes associated with cholesterol metabolism are known to be dysregulated both in CVD patients and patients with mCRPC [48,51].

*Arrhythmia*: Prolonged QT interval time is usually recognized as an increased risk factor for ventricular arrhythmia and sudden cardiac death [52,53]. In echocardiography/echocardiogram (ECG), the QT interval time starts from the beginning of the QRS complex (Q wave) and lasts till the end of the T wave. It is the duration of activation and recovery of the ventricular myocardium. Studies indicate that sex hormones, both endogenous and exogenous, have profound effects on QT interval time. Men usually have shorter QT intervals than women [47,52]. The reason is that testosterone shortens the QT interval, while estrogen has the opposite effect. It has been reported that men receiving ADT are at increased risk of developing torsades de pointes (TdP), QT prolongation, and ventricular arrhythmia [54]. ADT also interferes with QT interval time [47,52], and the development of arrhythmia in patients, as ADT inhibits testosterone production (abiraterone acetate) or interferes with testosterone function (enzalutamide). Abiraterone treatment has been shown to be associated with atrial tachyarrhythmia and heart failure, as abiraterone induces hypermineralocorticism [55]. Prolonged atrial tachycardia induces cardiomyopathy and, therefore, heart failure in patients.

In addition to the above-mentioned CVDs, ADT has also been linked to metabolic alterations like insulin resistance, obesity, and dyslipidemia which collectively increase CVD risk in PCa. ADT has also been reported to cause endothelial dysfunctions, which lead to vascular abnormalities resulting in the development of peripheral arterial diseases (PAD) like ischemic heart and limb ischemia [56,57,58] in patients.

## 4. Molecular Link between Cardiovascular Diseases and Prostate Cancer

To reveal the molecular link between CVDs and PCa, the RNAseq gene-level counts raw data were extracted from gene omnibus (GEO) (GSE126078, GSE80609) [46,59]. We analyzed the tumor tissue RNAseq data of mCRPC patients. RNAseq data of benign prostatic hyperplasia (BPH) patients served as control. The DESeq R package (1.24.0) [60,61] and iDEP.96 (http://bioinformatics.sdstate.edu/idep96/, accessed on 30 January 2023) [62] were used to perform differential gene expression analysis. Benjamini and Hochberg’s false discovery rate (FDR) was used to adjust the resulting *p*-values [63]. Genes with an adjusted *p*-value < 0.05 found by DESeq2 were considered as differentially expressed. The *p*-value was adjusted using the *q*-value [64]. The *q*-value < 0.05 and log2 (fold change) ≥ 2 were set as the threshold for significant differential expression by default. The Gene Ontology (GO) enrichment analysis of differentially expressed genes was implemented using iDEP.96 web-based analysis software. Finally, a protein-protein interaction (PPI) system of differentially expressed genes (DEGs) was formulated from the top 20 upregulated genes with the STRING database (version 11.5) [65].

**Figure 1:**
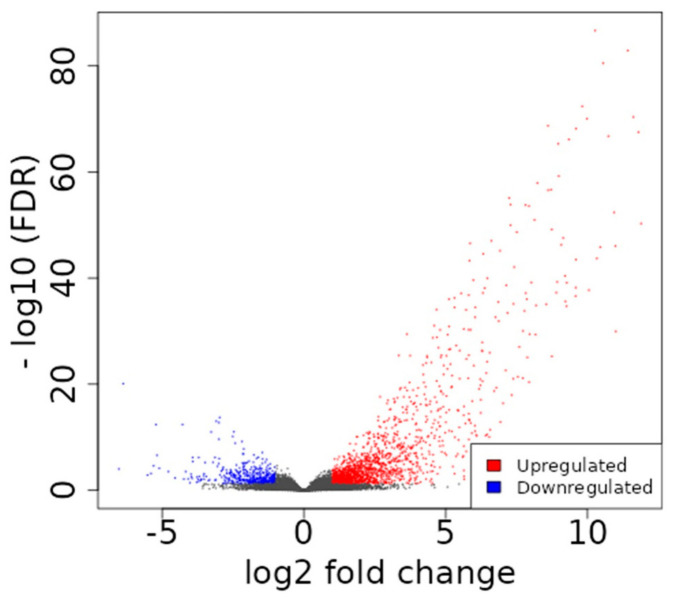
The volcano plot depicts the differential gene expression in CRPC vs. Control. The blue dots denote downregulated gene expression, and the red dots denote upregulated gene expression with fold threshold ≥ 2.0 and *p*-value < 0.05.

**Figure 2:**
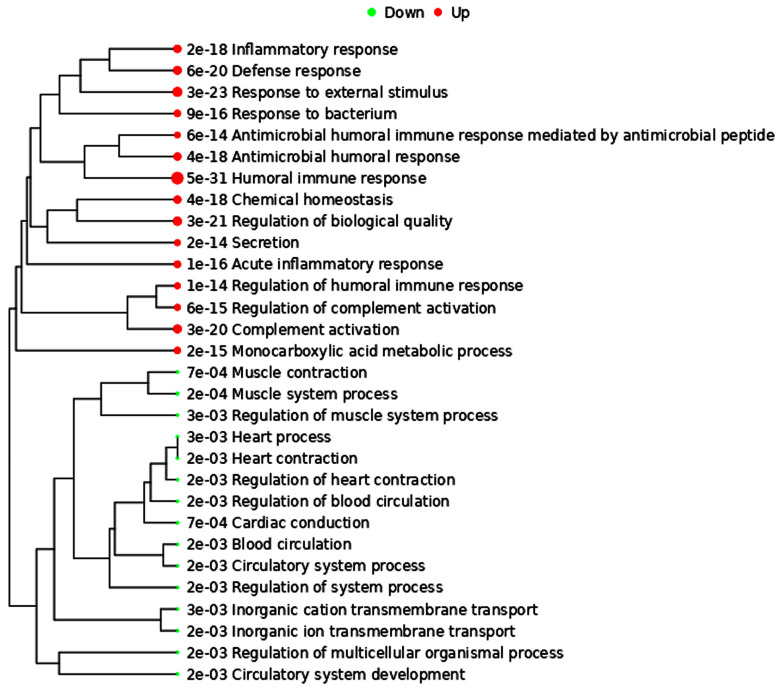
Gene ontology classification of genes according to biological processes. Red dots denote upregulation, and green dots denote downregulation.

Of the 1631 upregulated and 412 downregulated genes identified in mCRPC patients (Figure 1), the genes associated with cardiovascular processes (Figure 2) were found to be mostly downregulated. Downregulation was seen in *DES*, *ACTC1*, *OR51E2*, *CACNA1D*, *TBX18*, *PLN*, and *CASQ2* genes that play essential roles in the proper functioning of the cardiovascular system.

The *desmin (DES) gene* is primarily expressed in muscle tissues and plays vital roles in myocyte development, degeneration, and cellular functions [66]. It is a type III intermediate filament protein that helps maintain the structure and cytoskeletal organization of the striated heart muscle. Lack of desmin has profound effects on cardiac muscles. Mutated desmin or desmin deficiency has been linked to cardiac contractile dysfunction and impaired myocardial metabolism, which is a major reason for heart failure. Desmin deficiency also impacts the cardiac conduction system associated with familial cardiomyopathy [67]. In a meta-analysis performed in 2011, desmin mutation or lack of desmin function was found to be associated with increased cardiomyopathy, cardiac conduction disease or arrhythmia and atrioventricular blockage (AVB) [68].

*Cardiac actin gene 1 (ACTC1)* has been linked to hypertrophic cardiomyopathy (HCM) development. *ACTC1* encodes cardiac muscle alpha-actin, a major protein of the thin filament in cardiac sarcomeres responsible for muscle contraction and force generation to support the heart’s pump function [69]. Mutation or downregulation of *ACTC1* has been linked to dilated cardiomyopathy, the most frequent form of cardiomyopathy. Furthermore, nonsynonymous *ACTC1* mutation (p. G247D) is associated with a combined, unique phenotype of familial atrial septal defect (ASD-II) and dilated cardiomyopathy (DCM) [70]. *ACTC1* mutation was also detected in patients with left ventricular noncompaction (LVNC), repeated syncope, and resuscitated ventricular arrhythmias. It has been shown, particularly in LVNC patients, that this mutation could be related to potentially fatal arrhythmias [71].

*Olfactory receptor 51E2 (OR51E2)* encoded protein is suppressed in mCRPC patients. *OR51E1*, a paralog of *OR51E2*, is known to be associated with inotropic action in cardiac trabeculae and plays an important role in slice preparations of human explanted ventricles [72].

*The calcium voltage-gated channel subunit alpha1 D (CACNA1D)* gene belongs to a gene family that directs the formation of calcium channels that transport calcium ions across cell membranes. The *CACNA1D* gene, which encodes the α1 subunit of the Cav1.3 L-type calcium channel, effectively regulates intracellular Ca^2+^ stability. Several recent clinical studies have shown that *CACNA1D* polymorphisms are associated with hypertension [73,74,75].

*T-box transcription factor 18 (TBX18)*, gene encoded protein, TBX18, is a member of the T-box transcription factor family [76] and is primarily responsible for normal heart development. Loss of gene function studies have shown that *TBX1, TBX2, TBX3, TBX5, TBX18*, and *TBX20* play critical roles in cardiac development, ranging from cardiac lineage determination to vulvuloseptal development. *TBX18* has been shown to possess the repressor activity of SRF/CArG box, capable of inhibiting the progenitor cell differentiation into smooth muscle cells suggesting that *TBX18* has a potential function in maintaining the progenitor status of epicardial derived cells [77]. In addition, it is a potential candidate for gene therapy for pacemaker activity [78].

*Phospholamban (PLN)* encodes a 52-amino acid integral membrane protein that plays a major role in maintaining the calcium (Ca^2+^) pump in cardiac muscle cells [79]. It primarily inhibits sarco/endoplasmic reticulum Ca^2+^-ATPase (SERCA), which regulates calcium transport from the cytosol to the sarcoplasmic reticulum. *PLN* plays a key role in the maintenance of diastolic function, and mutation in this gene has been shown to cause dilated cardiomyopathy with refractory congestive heart failure [79,80]. Animal studies have confirmed the role of *PLN* in heart failure. Homozygous deletion of *PLN* is associated with an accelerated phenotype, which includes all forms of cardiac complications like contractile dysfunction, reduced ECG potentials, and cardiac dilation, and has a high susceptibility to ex vivo-induced arrhythmias and myocardial fibrosis [81].

*Calsequestrin 2 (CASQ2)* gene encodes the most abundant protein found in the sarcoplasmic membrane of cardiomyocytes which shows low affinity but high capacity binding to intracellular calcium [82]. In addition, it also plays a major role in calcium handling and release from the sarcoplasmic reticulum channel. Mutations in the *CASQ2* gene are associated with ventricular arrhythmia. Early mortality with dysfunctional cardiovascular events such as cardiac hypertrophy and contractile dysfunction have been reported in mice lacking the expression of *CASQ2* [83].

**Figure 3:**
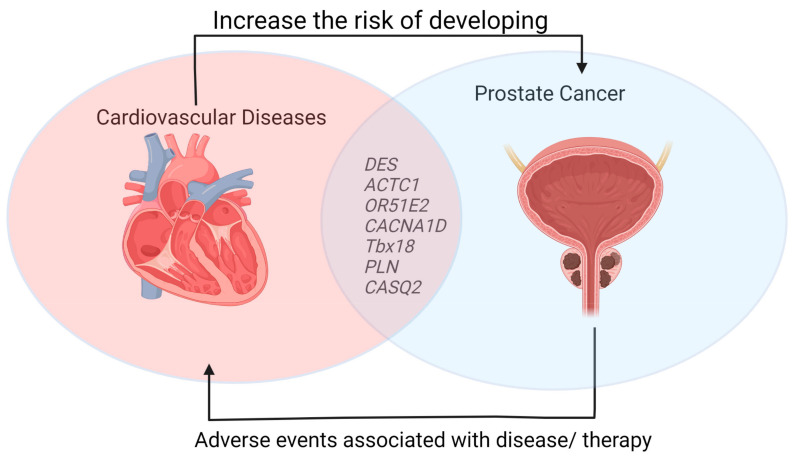
A molecular link between prostate cancer and cardiovascular diseases (Created by BioRender).

A schematic diagram showing the probable molecular link between the CVDs and PCa is represented in Figure 3.

**Figure 4:**
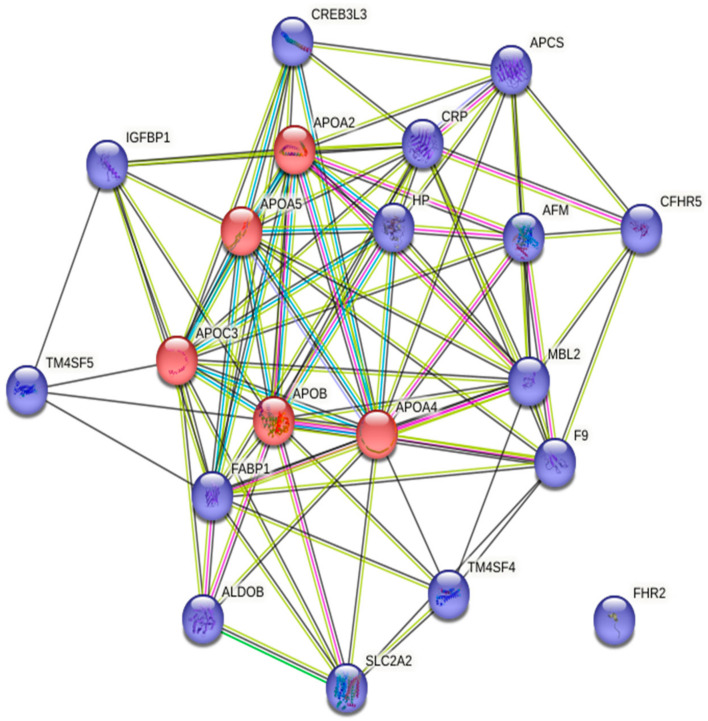
Protein-protein interactions (PPI) among the top 20 upregulated genes retrieved from the STRING database. Genes belonging to the apolipoprotein family are in red.

In addition to these cardiac-related genes, gene ontology analysis revealed that pathways enriched by the differentially expressed genes in mCRPC patients are mostly associated with cholesterol metabolism, bile secretion, and complement and coagulation cascade, which play an important role in CVDs. Cholesterol metabolism and cholesterol homeostasis play major roles in developing atherosclerosis and other cardiovascular complications [84,85]. Increased plasma cholesterol levels have been shown to be associated with atherosclerosis [86]. Bile acids comprise the primary catabolic pathway of cholesterol metabolism in the body. Bile acids content is associated with intratumoral androgen signature and sustained AR signaling that promotes tumor growth [87]. Complement activation, precisely the generation of C5a and C5b-9, influences many processes involved in the development and progression of atherosclerosis like promoting endothelial cell activation, leukocyte infiltration into the extracellular matrix, stimulating cytokine release from vascular smooth muscle cells, and promotion of plaque rupture. Complement activation also influences thrombosis by activating platelets, promoting fibrin formation, and impairing fibrinolysis [88]. The protein-protein interactions, when analyzed through the STRING database (version 11.5) (Figure 4), showed that the five apolipoproteins (APOA2, APOA4, APOB, APOA5 and APOC3) that are responsible for transporting lipids throughout the lymphatic and circulatory systems and associated with neurodegenerative diseases and CVDs, are highly upregulated in mCRPC. The role of apolipoproteins in CVD has been well investigated, but their participation in cancer has only been explored in a few published studies. Malik et al. found overexpression of APOA2 in the serum of PCa patients [89]. APOA2 is the second most abundant protein in high-density lipoproteins (25% of protein mass) and is primarily synthesized by the liver [90], but its function is largely unknown in PCa. The association between APOA4, APOB, APOA5 and APOC3 and PCa has also not been examined thoroughly.

## 5. Cardiovascular Events Associated with Second-Generation ADTs: Results from Clinical Trials with CRPC Patients

This section will discuss the second-generation ADTs (Enzalutamide, Abiraterone acetate, Apalutamide and Darolutamide) and the various adverse cardiovascular events associated with their use. We have chosen only the Phase III and Phase IV trials done with CRPC patients. We have also only considered the trials with published results (Table 2).

*Enzalutamide (Xtandi)*: Enzalutamide is an oral AR inhibitor that competitively binds to androgens, prevents AR translocation from the cytoplasm to the nucleus and inhibits AR binding to chromosomal DNA [91]. The drug received its first food and drug administration (FDA) approval in 2012 for the treatment of mCRPC. However, it is also a therapeutic option for non-metastatic CRPC and metastatic hormone-sensitive PC (mHSPC). Enzalutamide significantly increases the overall survival (OS) of CRPC patients and the time to PSA progression, but serious AEs like angina pectoris, left bundle branch block, acute myocardial infarction, sinus bradycardia, coronary artery thrombosis, myocardial ischemia and hypertension were noted in these patients [92,93,94]. In a meta-analysis (n = 8660) to study the cardiovascular toxicity of enzalutamide in PCa, the incidence of all-grade and high-grade hypertension in the enzalutamide-treated group was 10.5% and 4.8%, respectively, compared to 4.2% (all-grade) and 2.2% (high-grade) in the placebo-treated control group [11]. The adverse cardiovascular effects seen with the administration of Enzalutamide are included in Table 2.

*Abiraterone acetate (Zytiga)*: Abiraterone acetate is an orally active acetate ester form of the steroidal compound abiraterone, usually given to PCa patients nonresponsive to other hormone therapies. The drug received its first FDA approval in 2011 for the treatment of men with mCRPC who have received prior chemotherapy containing docetaxel in combination with prednisone. Abiraterone acetate is converted to abiraterone that inhibits 17α-hydroxylase/C17, 20-lyase (CYP17), which reduces both androgen and corticosteroid synthesis, leading to a compensatory increase in adrenocorticotropic hormone and mineralocorticoid synthesis [91]. It effectively prolongs the OS of mCRPC patients who previously received chemotherapy [95]. Abiraterone treatment, however, increases the risk of hypertension [11] and heart failure [55]. The adverse cardiovascular effects seen in clinical trials with the administration of Abiraterone acetate are included in Table 2.

*Apalutamide (ARN-509, JNJ-56021927, Erleada)*: Apalutamide is an oral AR inhibitor that binds directly to the ligand binding domain of AR and inhibits its translocation to the nucleus, DNA binding and impedes AR-mediated transcription [91]. In 2018, it was approved by FDA for the treatment of patients with non-metastatic castration-resistant PCa. In 2019, it was also approved for patients with metastatic castration-sensitive PCa. Apalutamide treatment improves progression-free survival as well as the OS of patients. The cardiovascular events observed with Apalutamide have been summarized in Table 2.

*Darolutamide (ODM-201, BAY1841788, Nubeqa)*: Darolutamide is a potent AR inhibitor that inhibits testosterone-induced nuclear translocation of AR. It also blocks the activity of the tested mutant ARs arising in response to antiandrogen therapies and improves OS among patients with non-metastatic, castration-resistant PCa and mHSPC. FDA first approved it in 2019 for the treatment of patients with non-metastatic CRPC [91]. Darolutamide prolongs the metastasis-free survival of PCa patients [96,97]. The cardiovascular events observed with Darolutamide have been summarized in Table 2.

**Table 2:** Cardiovascular events observed with the second-generation ADTs.

Drug	Trial Number and Description	Status	Phase	Intervention	Enrollment	Cardiovascular Events	References
**Enzalutamide**	NCT00974311 (AFFIRM study; multinational randomized, double-blind, placebo-controlled)	Start date: 30 September 2009 End date: 2 November 2017	III	Arm 1: Enzalutamide Arm 2: Placebo	1199 participants	Hypertension (6.6% enzalutamide vs. 3.3% placebo)	[98]
	NCT01212991(PREVAIL study; multinational randomized, double-blind, placebo-controlled)	Start date: 16 September 2010 End date: 14 February 2019	III	Arm 1: Enzalutamide Arm 2: Placebo	1717 participants	Atrial fibrillation (1.15% enzalutamide vs. 0.71% placebo) Acute myocardial infarction (1.26% enzalutamide vs. 0.00% placebo) Hypertension (0.46% enzalutamide vs. 0.00% placebo)	[99]
	NCT02116582(multi-center, single arm)	Start date: 23 May 2014 End date: 29 September 2017	IV	Arm 1: Enzalutamide	215 participants	Cardiac failure (1.40% enzalutamide treated group)	[100]
	NCT01995513 (PLATO trial randomized, double-blind, placebo-controlled)	Start date: 22 October 2013 End date: 31 August 2022	IV	Arm 1: Enzalutamide + Abiraterone + Prednisone Arm 2: Placebo + Abiraterone + Prednisone	509 participants	Acute coronary syndrome (0.59% enzalutamide and 0.80% enzalutamide + abiraterone + prednisone vs. 0.00% placebo + abiraterone + prednisone) Acute myocardial infarction (0.39% enzalutamide and 0.80% enzalutamide + abiraterone + prednisone vs. 0.00% in placebo + abiraterone + prednisone) Atrial fibrillation (0.59% enzalutamide vs. 0.00% in enzalutamide + abiraterone + prednisone treated group as well as placebo + abiraterone + prednisone) Congestive cardiac failure (0.39% enzalutamide and 0.80% enzalutamide + abiraterone + prednisone *p* vs. 0.00% placebo + abiraterone + prednisone) Hypertension (0.20% enzalutamide and 0.80% enzalutamide + abiraterone + prednisone *p* vs. 0.00% placebo + abiraterone + prednisone)	[101]
**Abiraterone acetate**	NCT00887198(randomized, double-blind, placebo-controlled)	Start date: 28 April 2009 End date: 25 May 2017	III	Arm 1: Placebo + prednisone Arm 2: Abiraterone acetate + prednisone	1088 participants	Atrial fibrillation (2.03% abiraterone + prednisone vs. 1.48% placebo) Angina pectoris (1.11% abiraterone + prednisone vs. 0.19% placebo) Coronary artery disease (0.74% abiraterone + prednisone vs. 0.19% placebo) Myocardial infarction (0.74% abiraterone + prednisone vs. 0.93% placebo) Myocardial ischemia (0.37% abiraterone + prednisone vs. 0.00% placebo) Supraventricular tachycardia (0.37% abiraterone + prednisone vs. 0.00% placebo) Hypertension (23.80% abiraterone + prednisone vs. 13.52% placebo)	[102]
	NCT01715285(LATITUDE study; randomized, double-blind, placebo-controlled)	Start date: 12 February 2013 End date: 13 February 2022	III	Arm 1: Abiraterone acetate + Prednisone + ADT Arm 2: Placebo + ADT	1209 participants	Acute coronary syndrome (0.84% abiraterone acetate + prednisone + ADT vs. 0.00% placebo + ADT), Angina pectoris (0.50% abiraterone acetate + prednisone + ADT vs. 0.00% placebo + ADT) Myocardial infarction (0.50% abiraterone acetate + prednisone + ADT vs. 0.00% placebo + ADT) Cardiac failure (0.50% abiraterone acetate + prednisone + ADT vs. 0.00% placebo + ADT) Atrial fibrillation (0.34% abiraterone acetate + prednisone + ADT vs. 0.00% placebo + ADT) Hypertension (38.36% abiraterone acetate + prednisone + ADT vs. 22.09% placebo + ADT)	[103]
	NCT00638690 (randomized, double-blind, placebo-controlled)	Start date: May 2008 End date: October 2012	III	Arm 1: Abiraterone acetate plus prednisone/ prednisolone Arm 2: Placebo plus prednisone/prednisolone	1195 participants	Atrial fibrillation (0.76% abiraterone acetate vs. 0.76% placebo) Angina pectoris (0.38% abiraterone acetate vs. 0.00% placebo) Congestive cardiac failure (0.51% abiraterone acetate vs. 0.25% placebo) Myocardial infarction (0.88% abiraterone acetate vs. 0.25% placebo) Hypertension (9.99% abiraterone acetate vs. 6.85% placebo)	[104]
**Apalutamide**	NCT01946204(SPARTAN study; multicenter randomized, double-blind, placebo-controlled)	Start date: 14 October 2013 Estimated End date: 8 November 2023	III	Arm 1: Apalutamide Arm 2: Placebo	1207 participants	Atrial Fibrillation (0.87% apalutamide vs. 0.50% placebo) Cardiac Failure (0.37% apalutamide vs. 0.00% placebo) Congestive Cardiac Failure (0.50% apalutamide vs. 0.25% placebo)	[105,106]
	NCT02257736 (randomized, double-blind, placebo-controlled)	Start date: 26 November 2014 Estimated End date: 5 April 2023	III	Arm 1: Apalutamide + Abiraterone acetate + Prednisone Arm 2: Placebo + Abiraterone acetate + Prednisone	982 participants	Bradycardia (0.41% apalutamide + abiraterone acetate + prednisone vs. 0.00% placebo + abiraterone acetate + prednisone Myocardial Infarction (1.22% apalutamide + abiraterone acetate + prednisone vs. 0.20% placebo + abiraterone acetate + prednisone) Ventricular Tachycardia (0.41% apalutamide + abiraterone acetate + prednisone vs. 0.00% placebo + abiraterone acetate + prednisone)	[107]
**Darolutamide**	NCT02200614 (ARAMIS trial; multinational, randomized, double-blind, placebo-controlled)	Start date: 12 September 2014 End date: 14 June 2021	III	Arm 1: Darolutamide Arm 2: Placebo	1509 participants	Sudden cardiac death (0.1% darolutamide vs. 0.00% placebo) Cardiac failure (0.84% darolutamide vs. 0.72% placebo) Acute cardiac failure (0.10% darolutamide vs. 0.00% placebo) Congestive cardiac failure (0.10% darolutamide vs. 0.00% placebo) Coronary artery disease (0.21% darolutamide vs. 0.18% placebo) Atrial fibrillation (0.84% darolutamide vs. 0.54% placebo) Myocardial ischemia (0.21% darolutamide vs. 0.18% placebo) Bradycardia (0.21% darolutamide vs. 0.18% placebo)	[108]

## 6. Conclusions

Emerging reports have indicated the wide-ranging impact of PCa treatment strategies like ADT on patients’ risk of developing CVDs, like hypertension, CAD, arrhythmia and cardiac arrests [8,9,10,11,12,13,14]. Though the interconnection between cardiovascular health and PCa has been reported, the molecular mechanisms underlying this interconnection have not been completely explored. In this review, we have summarized the association between CVDs and PCa by analyzing genomic data from mCRPC patients and identifying and analyzing genes involved in cardiac processes, like cardiac conduction, maintenance of heart rhythm and pacemaker function that are differentially expressed in mCRPC. Additionally, we have identified the highly active biological pathways in mCRPC patients, like cholesterol metabolism and complement activation that can eventually lead to CVDs. We have also discussed the findings from clinical trials using ADT, which indicate the cardiovascular side effects of ADT and provide compelling evidence in support of the connection between PCa and CVD.

Since it is known that the ADT treatment in patients with pre-existing CVD confers a higher risk of exhibiting serious cardiac AEs, the cardiovascular health of these patients must be considered before the start of therapy and monitored periodically throughout the duration of treatment and after completion of therapy, as cardiac dysfunction can occur at any time and treatment with ADT lasting for years may lead to increased CVD risk over time. The field of cardio-oncology is evolving, and further research is needed for the development of a comprehensive cardio-oncology program for PCa patients that will enable physicians to make personalized recommendations for successful reduction of CVD through the selection of the optimal therapy based on the patient’s risk of developing CVD and ideal surveillance of patient’s cardiac health.

## Figures and Tables

**Table 1:** Adverse cardiovascular events observed with the use of GnRH receptor agonists and antagonists.

Drugs	Trial Number and Description	Status	Phase	Intervention	Enrollment	Cardiovascular Events	References
1. Degarelix (GnRH Receptor Antagonist) 2. Leuprolide (GnRH Receptor Agonist)	NCT02663908 (PRONOUNCE study; multi-center, randomized, assessor-blind, controlled)	Start date: 19 April 2016 Estimated End date: 29 March 2021	III	Arm 1: Degarelix Arm 2: Leuprolide	545 participants	Chronic cardiac failure (1.09% Degarelix vs. 0.37% Leuprolide) Cardiac failure (0.36% Degarelix vs. 0.74% Leuprolide) Atrial flutter (0.73% Degarelix vs. 0.00% Leuprolide) Complete Atrioventricular block (0.73% Degarelix vs. 0.00% Leuprolide) Bradycardia (0.73% Degarelix vs. 0.00% Leuprolide) Supraventricular tachycardia (0.36% Degarelix vs. 0.00% Leuprolide) Hypertension (6.18% Degarelix vs. 8.55% Leuprolide)	[37,38,39]
1. Relugolix (GnRH Receptor Antagonist) 2. Leuprolide Acetate(GnRH Receptor Agonist)	NCT03085095 (HERO study; multinational, randomized, open-label, parallel-group)	Start date: 18 April 2017 Estimated End date: 26 November 2021	III	Arm1: Relugolix Arm 2: Leuprolide Acetate	1134 participants	Acute myocardial infarction (0.80% Relugolix vs. 0.32% Leuprolide) Cardio-respiratory arrest (0.00% Relugolix vs. 0.97% Leuprolide) Acute Cardiac failure (0.00% Relugolix vs. 0.32% Leuprolide) Cardiopulmonary failure (0.00% Relugolix vs. 0.32% Leuprolide) Hypertension (7.88% Relugolix vs. 11.69% Leuprolide)	[40,41]

## Data Availability

The publicly available datasets that were analyzed can be accessed at https://www.ncbi.nlm.nih.gov/geo/query/acc.cgi?acc=gse126078 (accessed on 30 January 2023), https://www.ncbi.nlm.nih.gov/geo/query/acc.cgi?acc=GSE80609 (accessed on 30 January 2023).
